# Research on Trajectory-Tracking Control System of Tracked Wall-Climbing Robots

**DOI:** 10.3390/s24010144

**Published:** 2023-12-27

**Authors:** Haoyan Zhang, Jiaqi Wu, Yang An, Pengshu Xie, Da Cui

**Affiliations:** School of Mechanical and Aerospace Engineering, Jilin University, Changchun 130025, China; haoyanz22@mails.jlu.edu.cn (H.Z.); wujq@jlu.edu.cn (J.W.); any@jlu.edu.cn (Y.A.)

**Keywords:** tracked wall-climbing robot, dynamics, trajectory tracking control, fuzzy logic, computed-torque control

## Abstract

Different from the vehicles and robots that move on the ground, complex and nonlinear track–wall interactions bring considerable difficulties to the accurate control of tracked wall-climbing robots due to the effect of gravity and adsorption. In this article, the authors propose a trajectory-tracking control system for tracked wall-climbing robots based on the fuzzy logic computed-torque control (FLCT) method. A key element in the proposed control strategy is to consider the adsorption force and gravity compensation based on the dynamic model. Validated via numerical simulations and experiments, the results show that the proposed controller can track the reference trajectory quickly, accurately and stably.

## 1. Introduction

**Figure 1:**
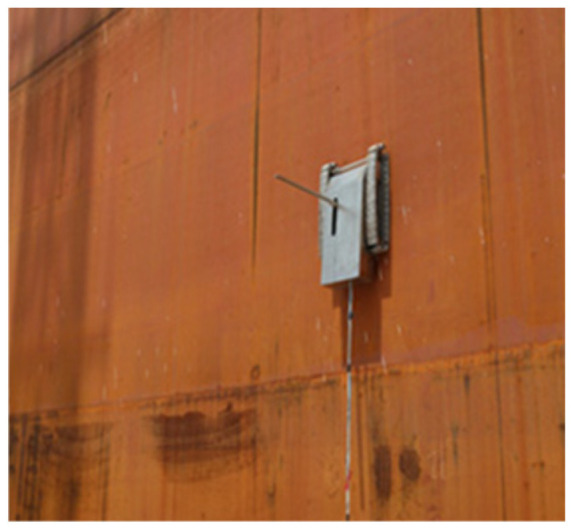
A typical application of a tracked wall-climbing robot [6].

Recently, the development of wall-climbing robots that can replace humans on vertical structures for technical applications, inspections, maintenance and construction tasks has gained huge attention. The earliest wall-climbing robot was built in 1986 and used crawlers as the walking mechanism [1]. Since then, after more than 60 years of development, various wall-climbing robots with different technologies have been developed for specific purposes [2]. Among them, the tracked wall-climbing robots combined with negative pressure adsorption technology show advantages in wall adaptability, moving speed, bearing capacity and stability. By installing sensors and working devices, dangerous tasks such as monitoring [3], inspection [4], welding [5], maintenance [6] and transportation [7] can be completed. Figure 1 shows a tracked wall-climbing robot used in ship inspection. However, only relying on human remote control has the problems of low accuracy and low efficiency. Therefore, it is necessary to develop an automatic motion control system for tracked wall-climbing robots.

Because it not only needs to have the same motion mechanism as traditional mobile robots but also must have the unique characteristics of being able to withstand gravity, tracked wall-climbing robots are influenced by nonlinear and strong coupling [8]. A PID controller is designed based on a kinematics model [5], which can automate welding tasks. However, the kinematics model of the tracked climbing robots is basically the same as their ground counterparts. Moreover, the tracked wall-climbing robot is a mechanical system with nonholonomic constraints. The characteristic of the nonholonomic system is that it has nonintegrable constraints and cannot express the position of the robot in the workspace according to the displacement of the driving parts. Despite that the simple controller based only on the kinematic model can simplify the tracking problem of a nonholonomic constraint system [9], it is based on the assumption that there is no slip, which is not in line with the actual situation. It can be noted that the dynamic model of the crawler wall-climbing robot must consider the influence of adsorption force and gravity. Therefore, designing the trajectory-tracking controller of the tracked wall-climbing robot must be based on the dynamic model, which includes the influence of force and uncertainties.

To stabilize the nonlinear system, Yoshio Katsuki et al. [10] proposed a trajectory-tracking controller for a wheeled wall-climbing robot by compensating for the nonlinear influences of gravity and attraction force. Experimental results show that the controller has a good compensation effect on gravity and gravity, and the trajectory quickly converges to the desired path. A sliding mode variable structure control has the advantages of fast response, insensitivity to parameter changes and disturbances, no online identification of the system and simple physical realization, because the sliding mode can be designed and has nothing to do with object parameters and disturbances. However, the disadvantage of this method is that when the state trajectory reaches the sliding surface, it is more difficult to slide strictly along the sliding surface toward the equilibrium point than to traverse back and forth on both sides of the sliding surface, resulting in chattering. To solve this problem, Lai Xin et al. [8] propose a back-stepping fuzzy adaptive sliding mode trajectory-tracking controller for a wheeled wall-climbing robot. However, the significant difference between the crawler and the wheel makes the wall motion control have to consider the effect of the steering resistance torque; otherwise, it will be difficult to steer.

The research on the automatic control of tracked vehicles and robots moving on the ground has become a focus of research interest. Based on the skid dynamics model, Hong et al. [11] proposed a path-tracking control algorithm for underwater tracked vehicles that takes into account the soil–track interaction. Wang et al. [12] designed a path-tracking controller tracked vehicle based on visual navigation and a fuzzy Proportional–Integral–Derivative (PID) algorithm. In this work, the accuracy of tracking control is not very satisfactory. To improve the accuracy of trajectory tracking, Zou et al. [13] proposed a novel approach to the motion control of tracked vehicles based on a modified PID computed-torque control. A key factor in this control strategy is to be able to reliably estimate the pose of the vehicle and its twist. In addition, Zhou et al. [14] designed a trajectory-tracking controller of tracked vehicles based on model predictive control using a linearized kinematics model. Experimental results based on visual navigation show a feasible control tracking effect [15]. Considering the sliding steering characteristics of tracked vehicles using the MPC method, the trajectory-tracking accuracy is improved by adjusting the weight coefficients of the objective function [16]. The online estimation and compensation of slip effects use an AutoDisturbance Rejection Controller (ADRC) to adapt to many different road conditions while improving path tracking accuracy. Sabiha A D et al. [17] proposed an optimized backpropagation controller as a kinematic controller to obtain the desired torque and converge its trajectory to the desired torque using vehicle dynamics and sliding characteristics of integral sliding mode control (SMC).

However, most of the current research on crawler robots focuses on improving the accuracy of trajectory tracking, and there are fewer studies on simultaneously improving tracking accuracy, as well as the effect of negative pressure control, and there is a lack of automatic control techniques for crawler wall-climbing robots that take into account the nonlinearities and couplings induced by gravity and adsorption forces. Computed-torque control (CTC) is a well-known motion control strategy for robotic systems [18]; it is geared towards the direct calculation of the required joint moments and can deal directly with the dynamics model and is therefore well adapted to systems that are nonlinear and coupled between joints. In addition, the method is able to compensate for adsorption force and gravity to feedforward systems and ensure global asymptotic stability. In this work, a trajectory-tracking controller is proposed based on computed-torque control for tracked wall-climbing robots undergoing skid steering on an inclined, hard wall. To overcome the disadvantage that the CTC design is subject to structured and unstructured uncertainties, fuzzy logic is adopted to compensate for these uncertain dynamics. The rest of this paper is arranged as follows: In Section 2, the force of the wall-climbing robot is analyzed, and the dynamic model with nonholonomic constraints is established. In Section 3, the controller design based on the computed-torque control method is introduced in detail, and the method of adjusting the parameters of the controller online by using fuzzy rules is given. In Section 4, the simulation analysis of trajectory tracking and negative pressure value controlling are carried out in MATLAB/Simulink, and the effect of the trajectory-tracking controller, as well as the negative pressure controller, is verified. The experiment is carried out in Section 5. Finally, Section 6 elaborates the conclusions.

## 2. Model Description

**Figure 2:**
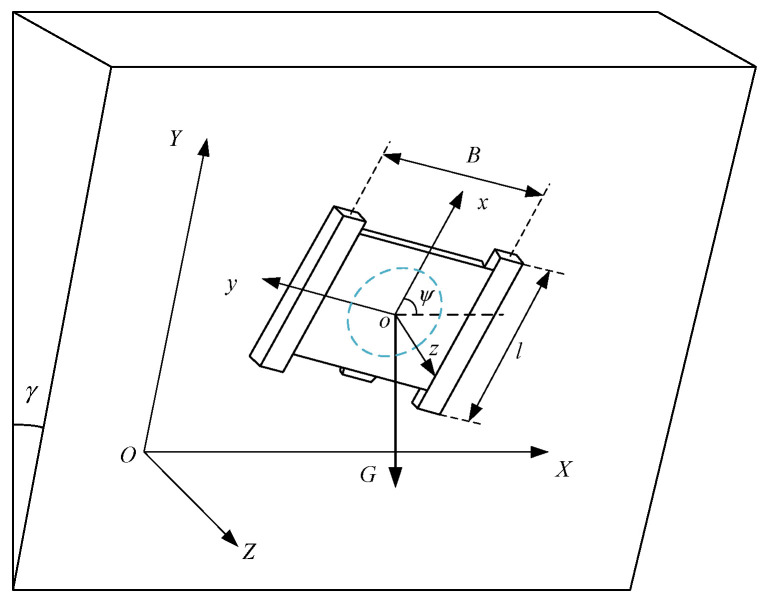
Coordinate system of the wall-climbing robot.

The crawler on both sides of the wall-climbing robot closely adheres to the wall under the action of the adsorption force *P* and moves against the gravity *G*. As shown in Figure 2, *oxyz* is the coordinate system fixed on the geometric center of the robot, and *OXYZ* is the inertial coordinate system. *Γ* and *ψ* are the inclination angle of the wall and the yaw angle of the robot, respectively.

The wall-climbing robot discharges the air in the negative pressure cavity by the negative pressure generating device and restricts the air from leaking into the negative pressure cavity from the outside through the sealing mechanism. The negative pressure is formed in the negative pressure chamber, so as to generate the adsorption force and make the robot adsorb on the wall.

**Figure 3:**
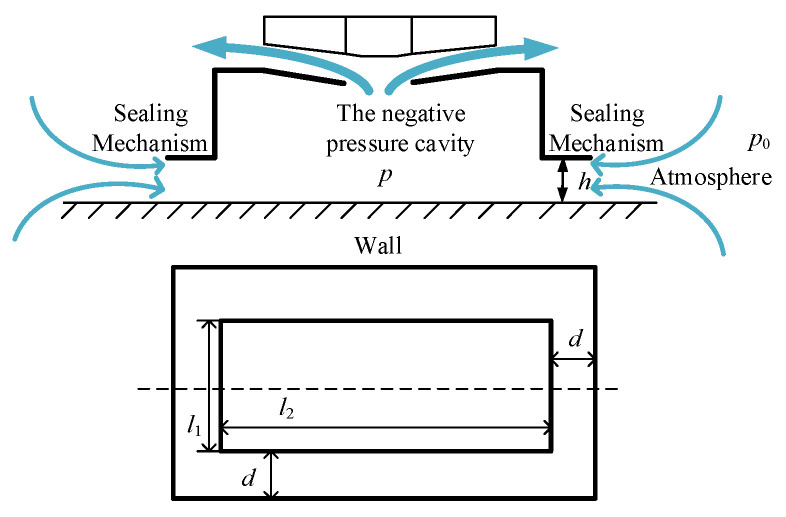
Sealing mechanism of the wall-climbing robot.

As shown in Figure 3, the air pressure outside the negative pressure chamber is *p*_0_, and after passing through the gap, the pressure changes to *p*, resulting in a linear pressure distribution with a pressure difference of $\Delta p=p_{0}-p$, then the pressure generated inside the negative pressure chamber is *F_p_*:

(1) $$F_{p}=\left[p_{0}-p\right]l_{1}l_{2}$$

where *l*_1_ and *l*_2_ are the lengths of the two sides of the negative pressure cavity. The suction force of the negative pressure caused by the pressure difference between the inside and outside of the sealing ring is *F_δ_*.

(2) $$F_{\delta }=\int_{0}^{d}\left[p+\frac{p_{0}-p}{d}x\right]\left(l_{1}+l_{2}+2d\right)dx$$

where *d* is the width of the sealing edge. Therefore, the resultant negative pressure adsorption force generated by the negative pressure system is

(3) $$P=F_{p}+F_{\delta }$$

The supporting force of the wall to the sealing edge is *F_Nq_*:

(4) $$F_{Np}=F_{\delta }$$

The supporting forces of the wall to Track 1 and Track 2 are

(5a) $$F_{N1}=\frac{F_{p}-G\sin\gamma }{2}-\frac{GH\cos\gamma \cos\psi }{B}$$

(5b) $$F_{N2}=\frac{F_{p}-G\sin\gamma }{2}+\frac{GH\cos\gamma \cos\psi }{B}$$

### 2.1. Kinematics

**Figure 4:**
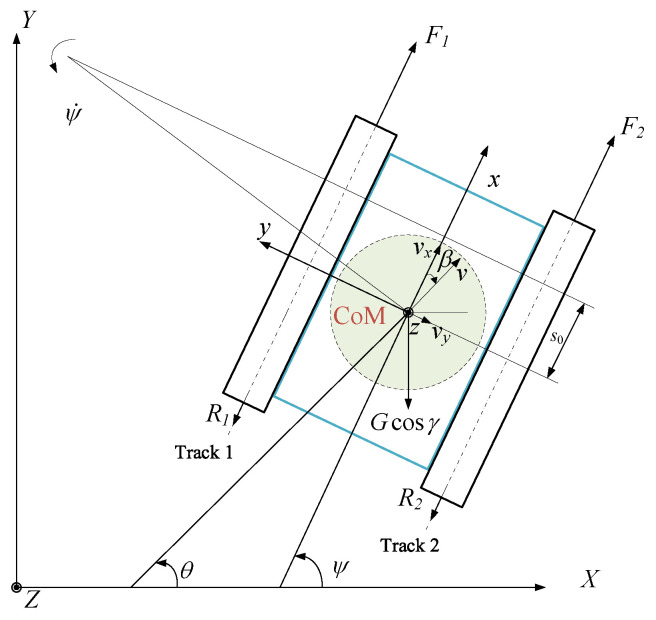
Wall motion diagram of the wall-climbing robot.

As shown in Figure 4, in the inertial coordinate system OXY, *v* is the linear velocity of the robot, and *θ* is the heading angle of the robot. The acceleration of the robot along the *x*-axis and *y*-axis can be expressed as

(6) $$\begin{array}{l} a_{x}={\dot{v}}_{x}-v_{y}\dot{\psi }\\ a_{y}={\dot{v}}_{x}+v_{y}\dot{\psi } \end{array}$$

The sideslip angle *β* can be calculated by [19]:

(7) $$\beta =\arctan\left(\frac{v_{y}}{v_{x}}\right)$$

The motion of the robot in an inertial coordinate system can be expressed as

(8) $$\left[\begin{array}{c} \dot{X}\\ \dot{Y}\\ \dot{\psi } \end{array}\right]=\left[\begin{array}{ccc} \cos\psi & -\sin\psi & 0\\ \sin\psi & \cos\psi & 0\\ 0 & 0 & 1 \end{array}\right]\left[\begin{array}{c} v_{x}\\ v_{y}\\ \dot{\psi } \end{array}\right]$$

Let *ω*_1_ and *ω*_2_ be the angular velocity of the sprockets and *r* be the pitch radius of the sprockets. The slips for the tracks on both sides can be calculated by

(9a) $$i_{1}=1-\frac{\dot{x}-\left(B/2\right)\dot{\psi }}{r\omega_{1}}$$

(9b) $$i_{2}=1-\frac{\dot{x}+\left(B/2\right)\dot{\psi }}{r\omega_{2}}$$

The velocity of the robot in the lateral direction is 0 at the point (*s*_0_, 0) in the *oxy* coordinates, the kinematic constraint at this point is

(10) $$\dot{Y}\cos\psi -\dot{X}\sin\psi -s_{0}\dot{\psi }=0$$

Taking $v={\left[v,\dot{\psi }\right]}^{T}$ as the control objectives, based on the kinematic constraints described in the above formula, the kinematics model of the robot can be expressed as

(11) $$\dot{q}=J(q)\dot{v}$$

where $J(q)=\left[\begin{array}{cc} \cos\psi & s_{0}\sin\psi \\ \sin\psi & -s_{0}\cos\psi \\ 0 & 1 \end{array}\right]$.

### 2.2. Dynamics

In the trajectory-tracking control of tracked robots, the angular velocities of the sprockets are generally used as the controller output variables. The friction generated by the relative movement between the tracks of the wall-climbing robot and the wall can be considered to comply with Coulomb’s law of friction [19,20]. Coulomb’s law of friction assumes that once the track and the wall have relative motion, the friction will reach the maximum value. The general theory of skid steering on firm ground proves that the shear stress depends on the shear deformation [21]. When the shear deformation modulus K is very small, the slip displacement–shear stress relationship will converge to simple Coulomb’s friction. Therefore, the relationship between the longitudinal tractive force of the track and the angular velocity of the sprocket of the wall-climbing robot can be established according to the general theory without reducing the accuracy, as long as the parameters are reasonable. Let F1 and F2 be the longitudinal tractive force of the tracks:

(12a) $$F_{1}=\mu F_{N1}\left[1-\frac{K}{i_{1}l}\left(1-e^{-i_{1}l/K}\right)\right]$$

(12b) $$F_{2}=\mu F_{N2}\left[1-\frac{K}{i_{2}l}\left(1-e^{-i_{2}l/K}\right)\right]$$

*F_y_* is the lateral resistance force of the track [22]:

(13) $$F_{y}=2\mathrm{sign}\left(\dot{\psi }\right)\mu_{t}s_{0}\frac{F_{N1}+F_{N2}}{l}$$

where *μ_t_* is the lateral resistance coefficient. The turning moment *M* generated by the longitudinal force is

(14) $$M=-\left(F_{1}-R_{1}\right)\frac{B}{2}+\left(F_{2}-R_{2}\right)\frac{B}{2}$$

The turning resistance moment caused by the lateral resistance can be expressed as [23]

(15) $$M_{r}=\mathrm{sign}\left(\dot{\psi }\right)\mu_{t}\frac{F_{N1}+F_{N2}}{l}\left(\frac{l^{2}}{4}-s_{0}{}^{2}\right)$$

The dynamics model of the wall-climbing robot is formulated in *xoy* as

(16a) $$m\ddot{x}=m\dot{y}\dot{\psi }+(F_{1}+F_{2})-(R_{1}+R_{2})-G\cos\gamma \sin\psi$$

(16b) $$m\ddot{y}=-m\dot{x}\dot{\psi }+F_{y}-G\cos\gamma \cos\psi$$

(16c) $$I_{z}\ddot{\psi }=M-M_{r}$$

Convert to inertial coordinate system:

(17a) $$m\ddot{X}=m\dot{Y}\dot{\psi }+(F_{1}+F_{2})\cos\psi -(R_{1}+R_{2})\cos\psi -F_{y}\sin\psi$$

(17b) $$m\ddot{Y}=-m\dot{X}\dot{\psi }+(F_{1}+F_{2})\sin\psi -(R_{1}+R_{2})\sin\psi +F_{y}\cos\psi -G\cos\gamma$$

(17c) $$I_{z}\ddot{\psi }=M-M_{r}$$

Reorganizing the above formulas can obtain the nonlinear dynamic model, including the nonholonomic constraints:

(18) $$M(q)\ddot{q}+F_{1}\left(q,\dot{q}\right)+F_{2}\left(q\right)+G\left(q\right)=B\left(q\right)\tau -A^{T}\left(q\right)\lambda$$

where $M(q)=\left[\begin{array}{ccc} m & 0 & 0\\ 0 & m & 0\\ 0 & 0 & I_{z} \end{array}\right]$, $F_{1}\left(q,\dot{q}\right)=\left[\begin{array}{c} -F_{y}\sin\psi \\ F_{y}\cos\psi \\ M_{r} \end{array}\right]$, $F_{2}\left(q\right)=\left[\begin{array}{c} \left(R_{1}+R_{2}\right)\cos\psi \\ \left(R_{1}+R_{2}\right)\sin\psi \\ \left(R_{1}-R_{2}\right)B/2 \end{array}\right]$, $G\left(q\right)=\left[\begin{array}{c} 0\\ G\sin\gamma \\ 0 \end{array}\right]$, $B\left(q\right)=\left[\begin{array}{cc} \cos\psi & \cos\psi \\ \sin\psi & \sin\psi \\ -B/2 & B/2 \end{array}\right]$, $\ddot{q}=\left[\begin{array}{c} \ddot{X}\\ \ddot{Y}\\ \ddot{\psi } \end{array}\right]$, $\tau =\left[\begin{array}{c} F_{1}\\ F_{2} \end{array}\right]$, $A^{T}\left(q\right)=\left[\begin{array}{c} -\sin\psi \\ \cos\psi \\ -s_{0} \end{array}\right]$,

λ is the constraint force. Differentiate Equation (11):

(19) $$\ddot{q}=\dot{J}\left(q\right)v\left(t\right)+J\left(q\right)\dot{v}\left(t\right)$$

Substitute Equation (19) into Equation (18):

(20) $$M(q)J\left(q\right)\dot{v}\left(t\right)+M(q)\dot{J}\left(q\right)v\left(t\right)+F_{1}\left(q,\dot{q}\right)+F_{2}\left(q\right)+G\left(q\right)=B\left(q\right)\tau -A^{T}\left(q\right)\lambda$$

Multiply both sides of the above formula to the left of matrix $J^{T}\left(q\right)$ to eliminate the constraint force:

(21) $$J^{T}MJ\dot{v}+J^{T}M\dot{J}v+J^{T}F_{1}+J^{T}F_{2}+J^{T}G=J^{T}B\tau$$

Rearrange the above formula:

(22) $$\bar{M}(q)\dot{v}+\bar{C}\left(q,\dot{q}\right)v+{\bar{F}}_{1}\left(q,\dot{q}\right)+{\bar{F}}_{2}\left(q\right)+\bar{G}\left(q\right)=\bar{B}\left(q\right)\tau$$

(23) $$\bar{\tau }=\bar{B}\left(q\right)\tau$$

where $\bar{M}(q)=J^{T}MJ=\left[\begin{array}{cc} m & 0\\ 0 & ms_{0}{}^{2}+I_{z} \end{array}\right]$, $\bar{C}\left(q,\dot{q}\right)=J^{T}M\dot{J}=\left[\begin{array}{cc} 0 & ms_{0}\dot{\psi }\\ -ms_{0}\dot{\varphi } & ms_{0}{\dot{s}}_{0} \end{array}\right]$, ${\bar{F}}_{1}\left(q,\dot{q}\right)=J^{T}C=\left[\begin{array}{c} 0\\ -F_{y}s_{0}+M_{r} \end{array}\right]$, ${\bar{F}}_{2}\left(q\right)=J^{T}F=\left[\begin{array}{c} R_{1}+R_{2}\\ \left(R_{1}-R_{2}\right)B/2 \end{array}\right]$, $\bar{G}\left(q\right)=J^{T}G=\left[\begin{array}{c} G\sin\gamma \sin\psi \\ -Gs_{0}\sin\gamma \cos\psi \end{array}\right]$, $\bar{B}\left(q\right)=J^{T}B=\left[\begin{array}{cc} 1 & 1\\ -B/2 & B/2 \end{array}\right]$.

### 2.3. Analysis of Safety Adsorption Conditions for Wall-Climbing Robots

The larger the adsorption force of the negative pressure adsorption system of the wall-climbing robot, the higher the stability of its movement on the wall. However, the flexibility of the robot’s movement will also decrease, and the power consumption of the motor will also increase. Therefore, determining the minimum adsorption force for the wall-climbing robot to work safely on the wall is conducive to improving the overall performance of the robot.

#### 2.3.1. Analysis of Slip Prevention Conditions

The wall-climbing robot slips because the friction between the tracks on both sides and between the bottom of the adsorption device and the wall cannot overcome the robot’s component of gravity along the wall direction. As shown in Figure 2, the wall-climbing robot’s force is balanced in the Y-axis direction:

(24) $$\mu F_{N1}+\mu F_{N2}+\mu_{\delta }F_{Nq}-G\cos\gamma =0$$

For the robot to not slip on the wall, the adsorption force generated by the negative pressure needs to be satisfied:

(25) $$\mu F_{p}+\mu_{\delta }F_{Nq}\geq G\cos\gamma +\mu G\sin\gamma$$

Assuming that the negative pressure adsorption force generated on the sealing ring is $1/k_{s}$ of the negative pressure adsorption force generated inside the negative pressure chamber, then the negative pressure difference Δp between the negative pressure chamber and the outside world should be satisfied:

(26) $$\Delta p=\frac{F_{p}}{A_{p}}\geq \frac{G\cos\gamma +\mu G\sin\gamma }{\left(\mu +\mu_{\delta }/k_{s}\right)A_{p}}$$

#### 2.3.2. Analysis of Tipping Prevention Constraints

When a wall-climbing robot works on the wall, a tendency to tumble around the contact point of the track and the wall is generated due to gravity. To ensure that the robot does not tumble on the wall in any attitude, the track plate under any support wheel should be made to contact the wall, i.e.,

(27) $$\min\left(p_{ij}\right)\geq 0$$

i=1,2,3,⋯n,j=1,2

Based on the load distribution study of the track, the dynamic load deflection due to acceleration is ignored at rest, and the center of mass and the center of negative pressure are assumed to coincide with the geometric center of the robot:

(28) $$\min\left(p_{ij}\right)=\frac{F_{p}}{2n}-\frac{G\sin\gamma }{2n}-\mathrm{sgn}(\cos\psi )\frac{GH\cos\gamma \cos\psi }{nB}-\mathrm{sgn}(\sin\psi )\frac{3\left(n-1\right)}{n\left(n+1\right)l}GH\cos\gamma \sin\psi \geq 0$$

Therefore, in order to prevent the robot from tipping over on the wall, the negative pressure difference between the inside and the outside of the negative pressure chamber should be satisfied:

(29) $$\Delta p=\frac{G\sin\gamma }{A_{p}}-\mathrm{sgn}\left(\cos\psi \right)\frac{2GH\cos\gamma \cos\psi }{BA_{p}}+\mathrm{sgn}(\sin\psi )\frac{2\left(n-1\right)}{A_{p}\left(n+1\right)l}GH\cos\gamma \sin\psi$$

#### 2.3.3. Straight-Line Motion Constraint Analysis

When the wall-climbing robot travels in a straight line on the wall, the friction between the tracks and the wall provides the driving force for the robot to move forward, and the forces are balanced in the direction along the longitudinal direction of the robot, and then there is

(30) $$F_{1}+F_{2}-G\cos\gamma \sin\psi -R_{1}-R_{2}-\mu_{\delta }F_{Nq}=ma_{x}$$

where $F_{1}$ and $F_{2}$ are the driving forces for the tracks on both sides, respectively.

The maximum thrust that can be generated by the tracks is limited by the nature of the wall and the parameters of the robot:

(31) $$\begin{array}{l} F_{1}\leq \mu F_{N1}\\ F_{2}\leq \mu F_{N2} \end{array}$$

In order for the robot to move straight on the wall, the negative pressure difference between the negative pressure chamber and the outside world should be satisfied:

(32) $$\Delta p\geq \frac{ma_{x}+G\cos\gamma \sin\psi +\mu_{\delta }F_{Nq}}{\left(\mu -\mu_{f}\right)A_{p}}-\frac{G\sin\gamma }{A_{p}}$$

#### 2.3.4. Steering Motion Constraint Analysis

When the wall-climbing robot steers and travels on the wall, the lateral friction between the tracks and the wall provides the driving force for the robot to steer, and the forces are balanced in the transverse direction along the robot, and then there are

(33) $$F_{1}^{\prime }+F_{2}^{\prime }-G\cos\gamma \cos\psi =ma_{y}$$

where $F_{1}^{\prime }$ and $F_{2}^{\prime }$ are the steering driving forces of the tracks on both sides, respectively.

In order for the robot to be able to steer and move on the wall without slipping, the negative pressure difference Δp between the negative pressure chamber and the outside world should be satisfied:

(34) $$\Delta p\geq \frac{G\cos\gamma \sin\psi }{\left(\mu^{\prime }+\mu_{\delta }^{\prime }/k_{s}\right)A_{p}}$$

In summary, the minimum safe negative pressure difference for the robot to move on the wall is

(35) $$\Delta p=\max\left\{\begin{array}{l} \frac{G\cos\gamma +\mu G\sin\gamma }{\left(\mu +\mu_{\delta }/k_{s}\right)A_{p}},\\ \frac{G\sin\gamma }{A_{p}}-\mathrm{sgn}\left(\cos\psi \right)\frac{2GH\cos\gamma \cos\psi }{BA_{p}}+\mathrm{sgn}(\sin\psi )\frac{2\left(n-1\right)}{A_{p}\left(n+1\right)l}GH\cos\gamma \sin\psi ,\\ \frac{ma_{x}+G\cos\gamma \sin\psi +\mu_{\delta }F_{Nq}}{\left(\mu -\mu_{f}\right)A_{p}}-\frac{G\sin\gamma }{A_{p}}\\ \frac{G\cos\gamma \sin\psi }{\left(\mu^{\prime }+\mu_{\delta }^{\prime }/k_{s}\right)A_{p}} \end{array}\right\}$$

**Figure 5:**
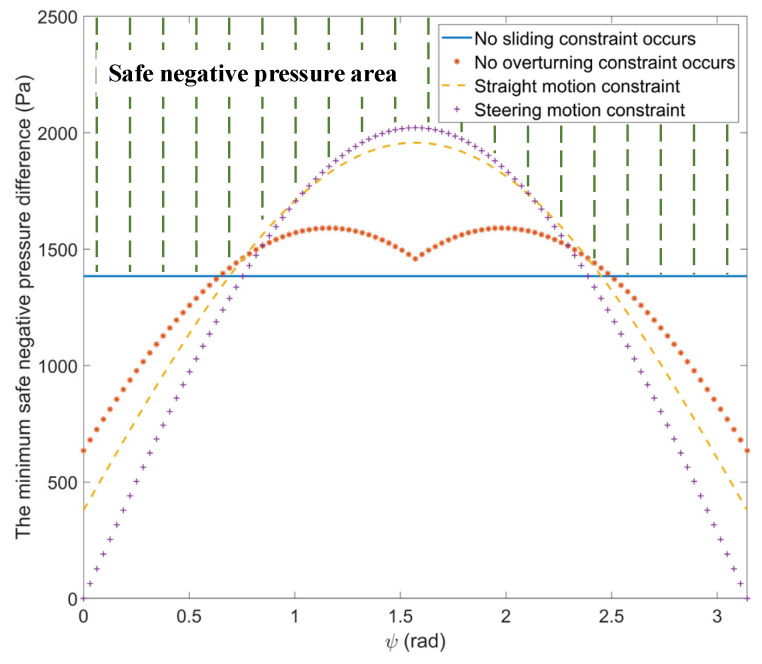
Safe negative pressure values for wall-climbing robots in different attitudes.

Substituting the parameters of the wall-climbing robot and the wall parameters into Equation (35) and setting the maximum longitudinal acceleration of the robot to 0.24 g, the minimum safe working negative pressure difference curve of the wall-climbing robot and the safe working area satisfying the constraints are shown in Figure 5.

## 3. Controller Design

### 3.1. Adaptive Negative Pressure Controller Design

The wall-climbing robot creates a negative pressure in the negative pressure chamber through the rotation of the fan to make the robot adsorb on the wall, so the robot controls the adsorption force generated by the negative pressure system by controlling the speed of the motor. When the adsorption force generated by the negative pressure system is too small, the robot’s motion mechanism will slip, and even lead to the robot slipping off the wall; and if the adsorption force is too large, although it can improve the safety of the robot’s motion, it will also make the walking resistance larger, increase the power consumption of the system, and cause excessive noise, steering difficulties and other problems. Therefore, in order to reduce the walking resistance and improve the driving efficiency of the robot while meeting the safety conditions, it is necessary to take the negative pressure of the adsorption system as one of the control variables of the trajectory tracking of the wall-climbing robot, so as to make the wall-climbing robot walk along the reference trajectory adaptively under the value of reasonable negative pressure.

When the wall-climbing robot travels on the wall, the negative pressure in the negative pressure chamber is adjusted by controlling the rotational speed of the centrifugal fan, and because the air leakage is constantly changing, the open-loop control cannot meet the demand for dynamic adjustment of the negative pressure. Therefore, a closed-loop negative pressure feedback control is used to realize the adaptive negative pressure adjustment during the trajectory tracking of the wall-climbing robot. First, according to the robot’s position, the minimum safe working negative pressure required in the current state of the robot is obtained based on Equation (35), which is multiplied by the safety factor $K_{s}$ as the desired negative pressure $p\left(\psi \right)$. The deviation between the actual negative pressure $p\left(t\right)$ and the desired negative pressure in the negative pressure chamber is input into the negative pressure controller as the control deviation. The control deviation is

(36) $$\delta_{p}=p\left(\psi \right)-p\left(t\right)$$

The expression of the negative pressure controller is

(37) $$n(s)=K_{f}p\left(\psi \right)+K_{pf}\delta_{p}+K_{if}\int_{0}^{t}\delta_{p}dt+K_{df}\frac{d\delta_{p}}{dt}$$

where n(s) is the fan speed, $K_{f}$ is the fan speed and the negative pressure generated by the proportionality coefficient and $K_{pf}$, $K_{if}$ and $K_{df}$ are fan control proportionality coefficients, integral coefficients and differential coefficients, respectively.

### 3.2. Trajectory-Tracking Controller Design

#### 3.2.1. Deviation Calculation

**Figure 6:**
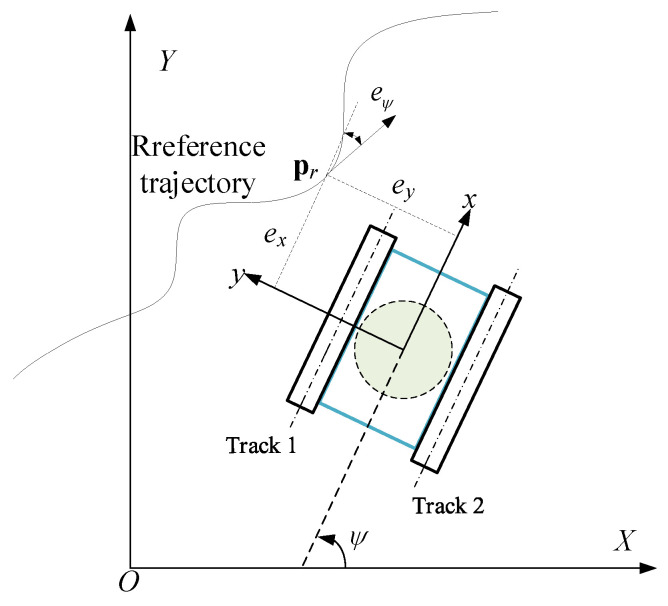
Trajectory deviation.

As shown in Figure 6, the deviation between the reference position $P_{r}={\left[\begin{array}{ccc} X_{r} & Y_{r} & \psi_{r} \end{array}\right]}^{T}$ and the actual position $P_{c}={\left[\begin{array}{ccc} X & Y & \psi \end{array}\right]}^{T}$ in the xoy coordinates is defined as the position deviation:

(38) $$e_{p}=\left[\begin{array}{c} e_{x}\\ e_{y}\\ e_{\varphi } \end{array}\right]=\left[\begin{array}{ccc} \cos\psi & \sin\psi & 0\\ -\sin\psi & \cos\psi & 0\\ 0 & 0 & 1 \end{array}\right]\left[\begin{array}{c} X_{r}-X\\ Y_{r}-Y\\ \psi_{r}-\psi \end{array}\right]$$

where *e_x_*, *e_y_* and $e_{\psi }$ are longitudinal deviation, lateral deviation and yaw angle deviation, respectively.

Differential Equation (24)

(39) $${\dot{e}}_{p}=\left[\begin{array}{c} {\dot{e}}_{x}\\ {\dot{e}}_{y}\\ {\dot{e}}_{\psi } \end{array}\right]=\left[\begin{array}{c} e_{y}\dot{\psi }-v+v_{r}\cos e_{\psi }\\ -e_{x}\dot{\psi }+v_{r}\sin e_{\psi }\\ \psi_{r}-\psi \end{array}\right]$$

The desired velocity of trajectory-tracking control of the robot is defined as

(40) $$v_{d}=\left[\begin{array}{c} v_{r}\cos e_{\psi }+K_{x}e_{x}\\ {\dot{\psi }}_{r}+v_{r}\left(K_{y}e_{y}+K_{\psi }\sin e_{\psi }\right) \end{array}\right]$$

(41) $$v_{d}=f\left(e_{p},v_{r},K\right),\;K={\left[\begin{array}{ccc} K_{x} & K_{y} & K_{\psi } \end{array}\right]}^{T}$$

where *K_x_*, *K_y_* and K*ψ* are gain coefficients. Differential $v_{d}$:

(42) $${\dot{v}}_{d}=\left[\begin{array}{c} {\dot{v}}_{r}\cos e_{\psi }-v_{r}{\dot{e}}_{\psi }\sin e_{\psi }+K_{x}{\dot{e}}_{x}\\ {\ddot{\psi }}_{r}+{\dot{v}}_{r}\left(K_{y}e_{y}+K_{\psi }\sin e_{\psi }\right)+v_{r}\left(K_{y}{\dot{e}}_{y}+K_{\psi }{\dot{e}}_{\psi }\cos e_{\psi }\right) \end{array}\right]$$

Define the twist-tracking error ***e****_v_* as

(43) $$e_{v}=v_{d}-v$$

**Figure 7:**
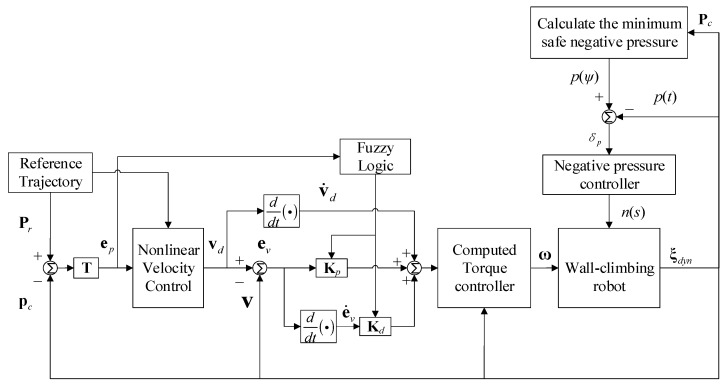
Control scheme of the trajectory tracking of the wall-climbing robot.

Based on the above definition, the proposed fuzzy logic computed-torque controller of the wall-climbing robot is

(44) $$\begin{array}{l} \bar{B}\tau =\bar{M}({\dot{v}}_{d}+K_{p}e_{v}+K_{d}{\dot{e}}_{v})+\bar{C}+{\bar{F}}_{1}+{\bar{F}}_{2}+\bar{G}\\ u={\bar{B}}^{-1}\bar{\tau } \end{array}$$

where $K_{p}=\mathrm{diag}(K_{p},K_{p})$ is the proportional gains matrix, and $K_{d}=\mathrm{diag}(K_{d},K_{d})$ is the differential gains matrix. Once the controller output u is obtained, the sprocket’s velocity $\omega ={\left[\omega_{1},\omega_{2}\right]}^{T}$ can be calculated by Formula (12). The gain parameters of the controller are obtained by online tuning according to the inference rules of the fuzzy system:

(45) $$\begin{array}{l} K_{y}=\left(K_{y\max}-K_{y\min}\right){K_{y}}^{\prime }+K_{y\min},\\ K_{\psi }=\left(K_{\psi \max}-K_{\psi \min}\right){K_{\psi }}^{\prime }+K_{\psi \min},\\ K_{p}=\left(K_{p\max}-K_{p\min}\right){K_{p}}^{\prime }+K_{p\min}. \end{array}$$

where $\left[K_{y\min},K_{y\max}\right]$, $\left[K_{\psi \min},K_{\psi \max}\right]$ and $\left[K_{p\min},K_{p\max}\right]$ are the ranges of lateral error gain coefficient, yaw angle deviation gain coefficient and proportional gain coefficient, respectively. The effect of the controller is not sensitive to the variation in the longitudinal deviation coefficient, so it is set as a constant. The differential gain coefficient *K_d_* can be selected according to *K_p_*, generally taken as *K_d_* = 0.1~0.001*K_p_* [24]. The overall trajectory-tracking controller block diagram of the wall-climbing robot is shown in Figure 7. In this model, the state output variables are selected as $\xi_{dym}={\left[X,Y,\psi ,\dot{\psi },\dot{x},\dot{y},\Delta p\right]}^{T}$, and the control quantity is selected as $\omega ={\left[\omega_{1},\omega_{2}\right]}^{T}$.

#### 3.2.2. Fuzzy Logic

**Figure 8:**
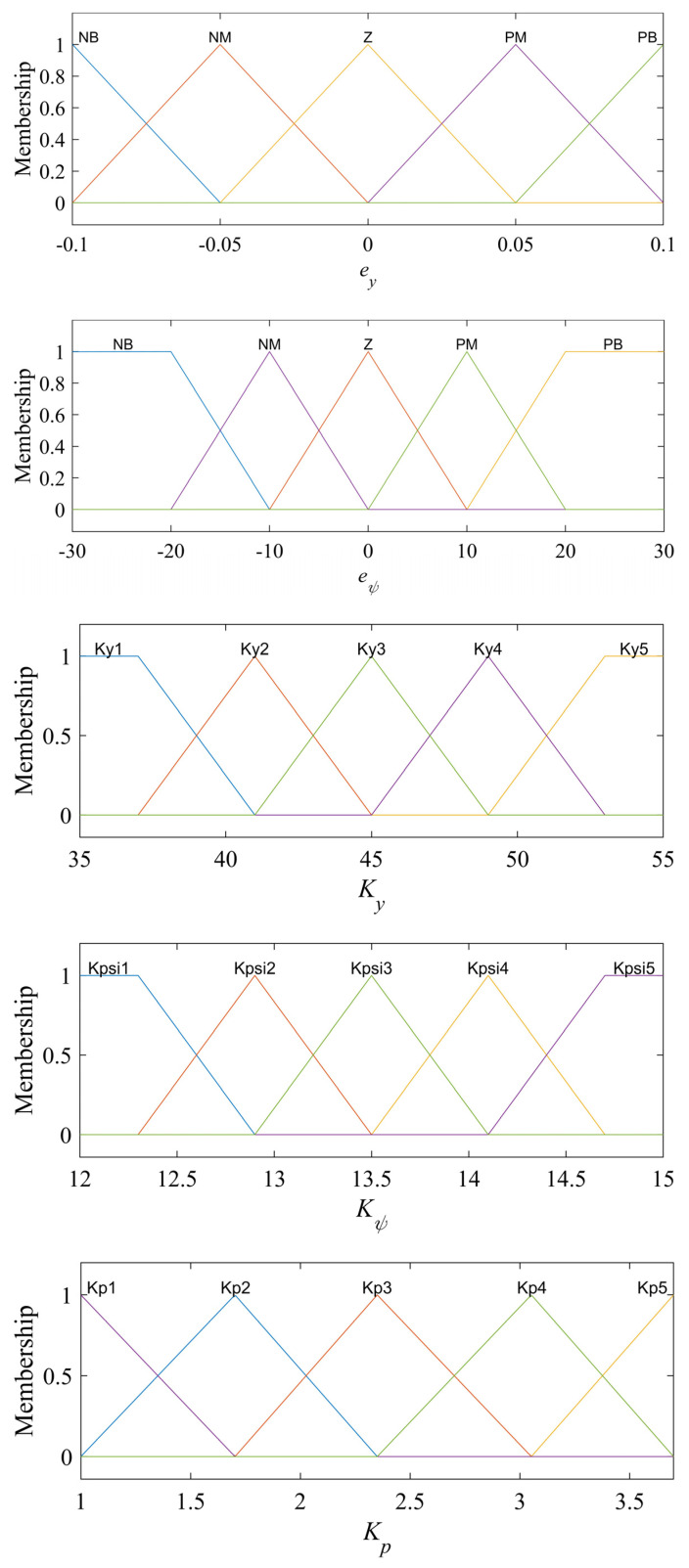
Membership function.

The input variables of the fuzzy logic system of the controller are the lateral deviation *e_y_* and the yaw angle deviation *e_ψ_*, and the output variables are the calculation of lateral deviation control coefficients in moment method controllers *K_y_*, control coefficient of swing angle deviation *K_ψ_* and proportional gain coefficient *K_p_*. The principle of the membership function between input variables and output variables is that when the lateral deviation is large, the controller can react quickly to make the robot give priority to reducing the lateral distance deviation; when the lateral deviation is reduced to a certain range, the controller can make the robot adjust in a smaller range to improve the accuracy. The control coefficients and proportional gain coefficients of the method for the trial method are generally determined, that is, according to the control system accuracy and speed requirements after several calculations to determine the best set of coefficients after comparison. The membership function of the fuzzy logic system is set according to experience and simulation tests. The membership functions of all inputs and outputs are shown in Figure 8.

**Figure 9:**
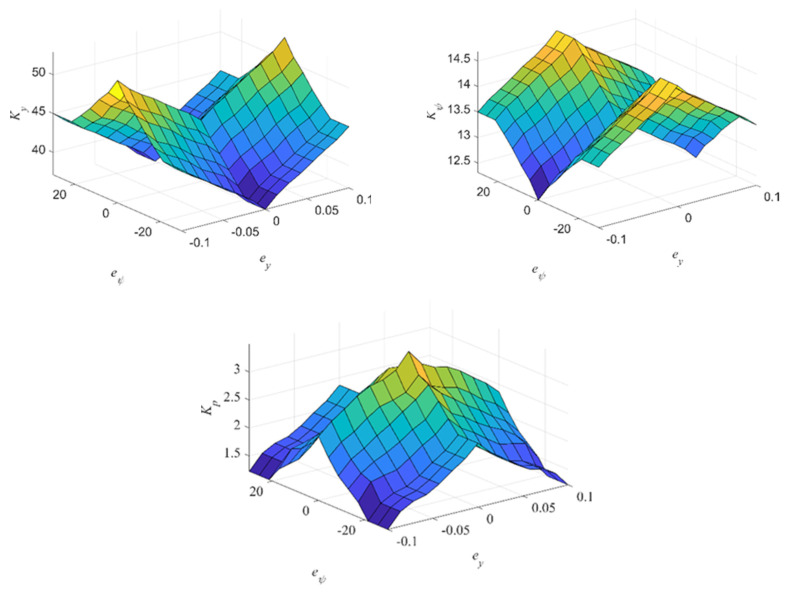
Fuzzy logic rule surface.

In order to improve the response and accuracy of the controller and quickly eliminate trajectory deviation, the fuzzy logic rules of the trajectory-tracking control system are shown in Table 1 and Figure 9:

1. When the lateral deviation is large, in order to enable the robot to quickly approach the reference trajectory, the gain parameter *K_y_* takes a larger value; when the lateral deviation is small, *K_y_* should be reduced to reduce the sensitivity of the controller to the lateral deviation to reduce the overshoot of the system. When the yaw angle deviation is large, *K_y_* takes a smaller value, so that the controller mainly takes the command to reduce the yaw angle deviation; when the yaw angle deviation is small, *K_y_* takes a larger value to improve accuracy with respect to the lateral deviation.
2. When the yaw angle deviation is large, in order to quickly adjust the driving direction of the robot, the yaw angle deviation control parameter *K_ψ_* should take a larger value; when the yaw angle deviation is small, *K_ψ_* should take a smaller value to reduce the overshoot of the yaw angle and avoid vibration. When the lateral deviation is large, *K_ψ_* is set to small to reduce the effect of yaw angle deviation; when the lateral deviation is small, *K_ψ_* should be increased to improve the control accuracy.
3. The parameter *Kp* can improve the response and accuracy of the controller [25]. *Kp* should be set to small when the lateral deviation and yaw angle deviation are large due to proportional coefficients potentially causing the system to respond quickly. When the lateral deviation and the yaw angle deviation are small, increase the *Kp* value to improve accuracy.

## 4. Simulations

**Figure 10:**
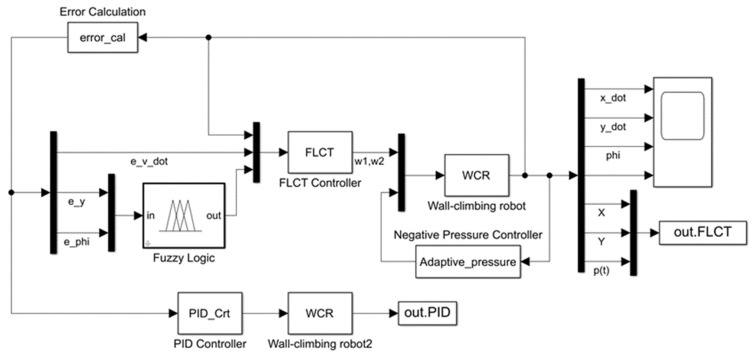
Matlab/Simulink simulation block diagram.

In order to verify the effectiveness of the proposed control algorithm, several simulations are performed in MATLAB/Simulink. The simulation model block diagram is shown in Figure 10, and the robot parameters and wall parameters are shown in Table 2. Compared with the robot’s dead weight and its ability to move, the connecting wires between the wall-climbing robot and the ground have a very small effect on the robot’s movement in real environments, so the grounded wire portion is ignored in the simulation.

The simulation scenarios include a straight-line reference trajectory and a circular reference trajectory. In order to verify the effect of the proposed fuzzy logic computed-torque control (FLCT) controller, it is compared with the classical PID controller. The classical PID controller is formed as follows:

(46) $$\left[\begin{array}{c} \omega_{1}\\ \omega_{2} \end{array}\right]=\left[\begin{array}{c} \frac{v_{r}}{r}+k_{ex}e_{x}-T_{p}\left(e_{\delta }+\frac{1}{T_{i}}\int_{0}^{t}e_{\delta }dt+T_{d}\frac{de_{\delta }}{dt}\right)\\ \frac{v_{r}}{r}+k_{ex}e_{x}+T_{p}\left(e_{\delta }+\frac{1}{T_{i}}\int_{0}^{t}e_{\delta }dt+T_{d}\frac{de_{\delta }}{dt}\right) \end{array}\right]$$

where *T_p_*, *T_i_* and *T_d_* are the proportional coefficient, integral time constant and derivative time constant of the PID controller, respectively, and *e_δ_* is the control deviation:

(47) $$e_{\delta }=e_{\psi }+\arctan k_{ey}\frac{e_{y}}{v}$$

### 4.1. Reference Straight Trajectory

**Figure 11:**
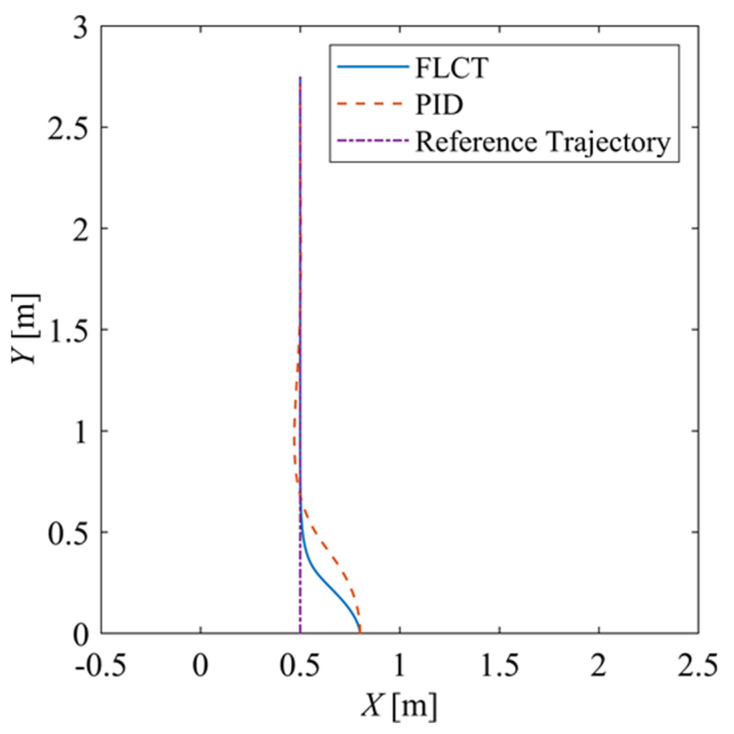
Trajectory of the XY-plane.

Figure 11 shows the motion trajectory. The reference straight trajectory has a length of 0.5 m, and it can be seen that the robot can quickly track the reference trajectory, reduce the deviation to basically 0 and drive stably along the reference trajectory. Compared with the PID control method, the FLCT controller has better performance.

**Figure 12:**
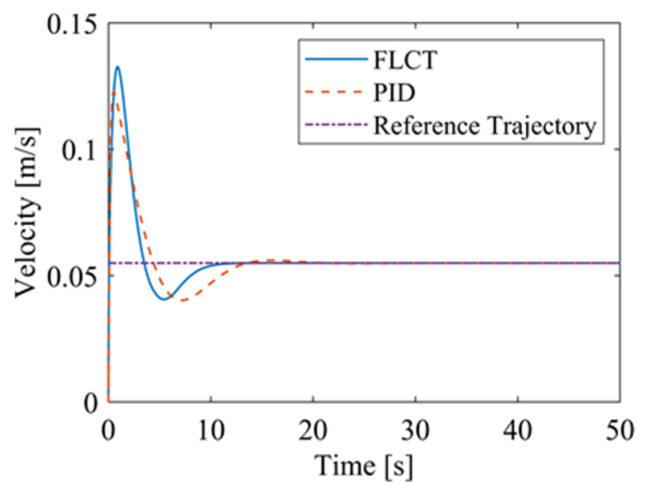
The velocity of the robot.

**Figure 13:**
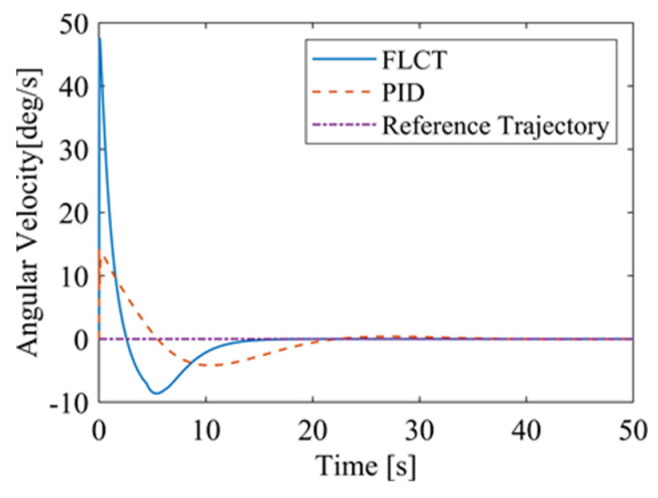
The angular velocity of the robot.

**Figure 14:**
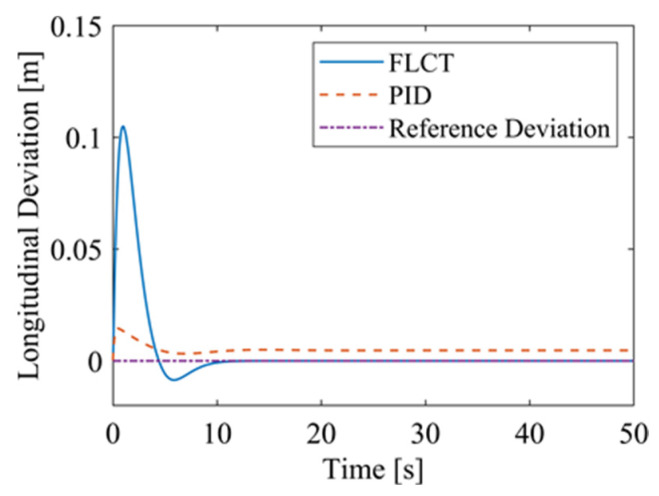
Longitudinal distance deviation.

**Figure 15:**
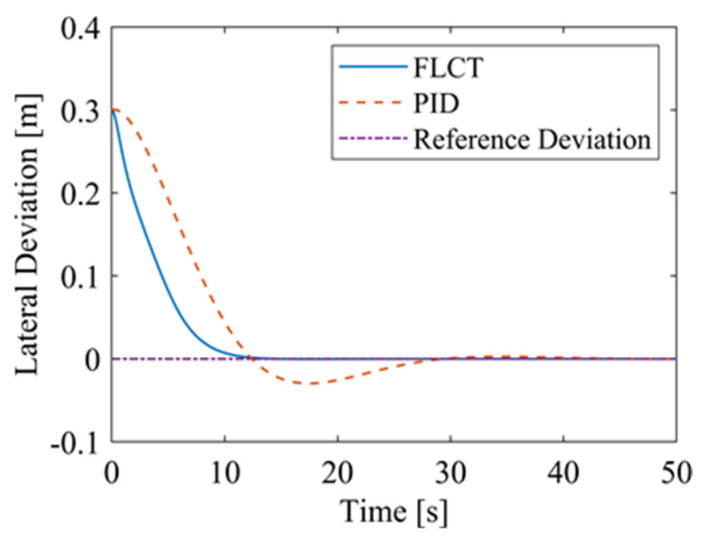
Lateral distance deviation.

**Figure 16:**
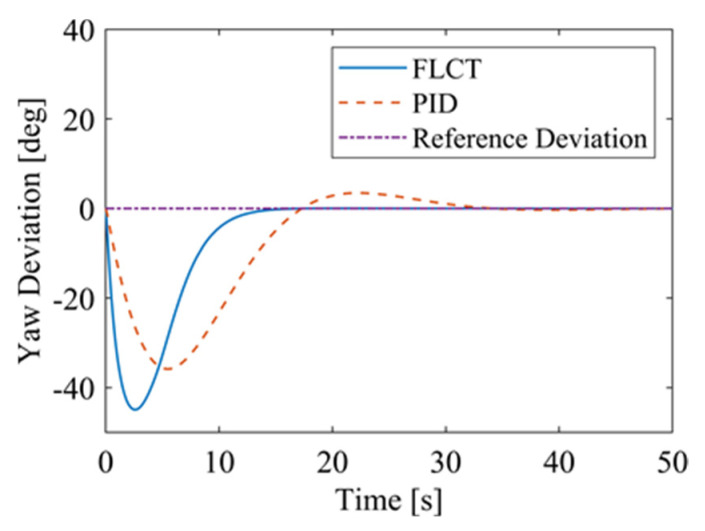
Yaw angle deviation.

**Figure 17:**
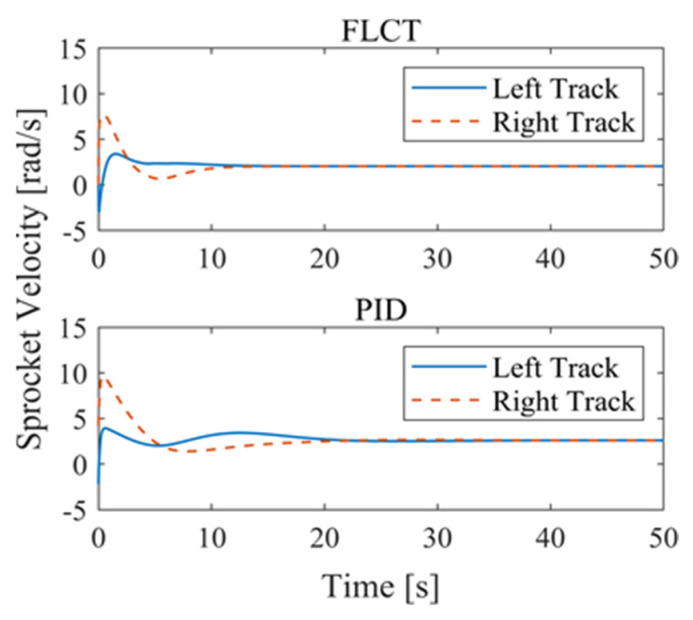
Sprocket velocities.

**Figure 18:**
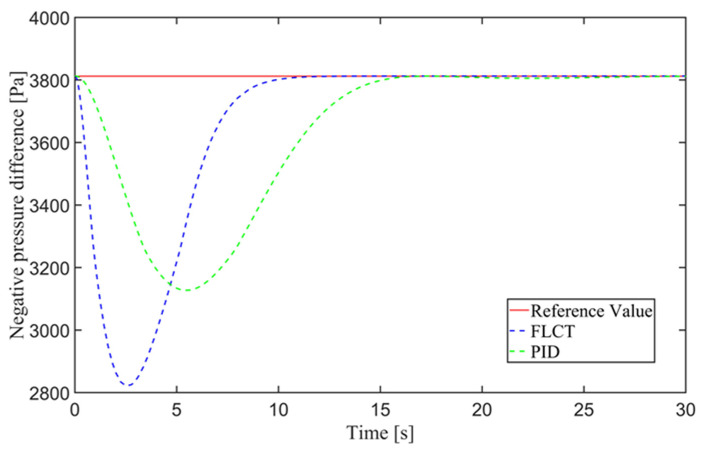
Negative pressure of the adsorption unit.

Figure 12 and Figure 13 show the change in the velocity and the angular velocity of the robot with time, where the vertical axes of Figure 12 and Figure 13 denote the traveling speed and the transverse pendulum angular velocity of the wall-climbing robot, respectively. For the FLCT controller, the velocities converge after 15 s, and then tend to stabilize. In order to observe the deviation of the trajectory tracking, Figure 14, Figure 15 and Figure 16 show the longitudinal distance deviation, lateral distance deviation and yaw angle deviation with time, where the vertical axis of Figure 14 and Figure 15 represents the longitudinal distance deviation value and the lateral distance deviation value between the actual trajectory and the ideal trajectory of the robot, respectively. For the FLCT controller, it can be seen from Figure 15 and Figure 16 that the initial lateral deviation is 0.2 m, and the yaw angle deviation is 0. At the initial stage, the controller is mainly to reduce the lateral distance deviation. At 1.2 s, the robot is close to the reference trajectory and the yaw angle deviation of the controller increases, causing the robot to gradually return to the correct trajectory, and finally, it is consistent with the reference trajectory. Compared with the PID controller, the setting time and the overshoot of FLCT controller are smaller. As shown in Figure 17, the angular velocity of the sprockets gradually converges as the deviation decreases. Figure 18 shows the variation in the negative pressure value within the adsorption system. In the initial stage of the movement, the negative pressure value changes rapidly with the robot’s attitude, and when the robot reaches a stable driving state, the negative pressure value also tends to stabilize. Compared with the PID controller, the FLCT controller reaches the stable state more quickly. The FLCT controller has a better control effect in terms of the time required to achieve stable control and the accuracy of the stable control.

### 4.2. Reference Curve Trajectory

**Figure 19:**
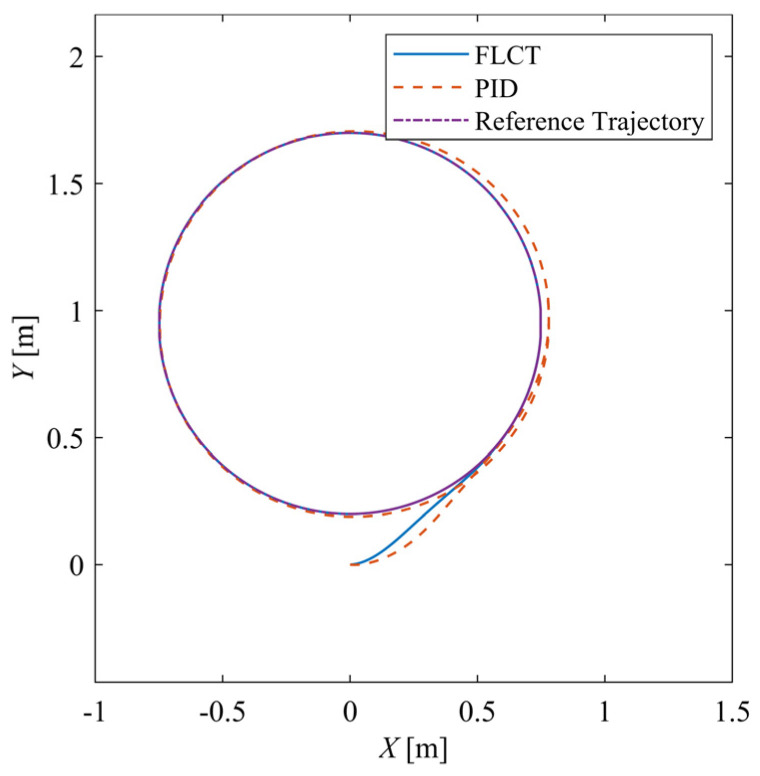
Trajectory of the XY-plane.

The reference curve trajectory is a circle with a diameter of 0.75 m, the reference velocity vr is 0.075 m/s and the reference angular velocity ${\dot{\psi }}_{r}$ is 0.1 rad/s. Figure 19 shows the trajectory of the robot. Similar to the result of the straight-line scenario, under the action of the FLCT controller, the robot can quickly track the reference trajectory and drive stably along the reference trajectory, which has a better control effect.

**Figure 20:**
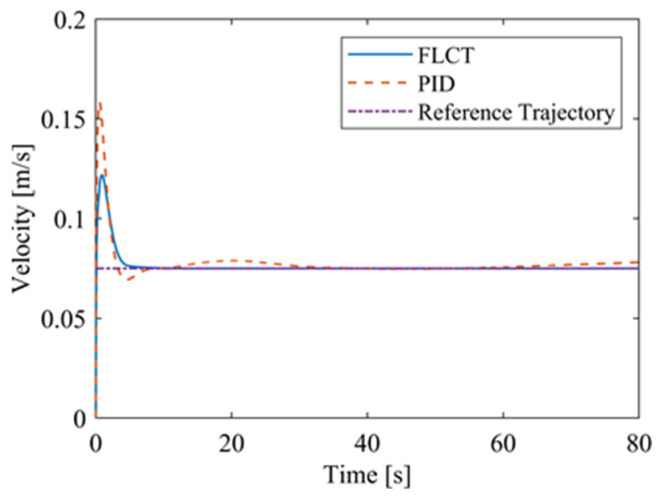
The velocity of the robot.

**Figure 21:**
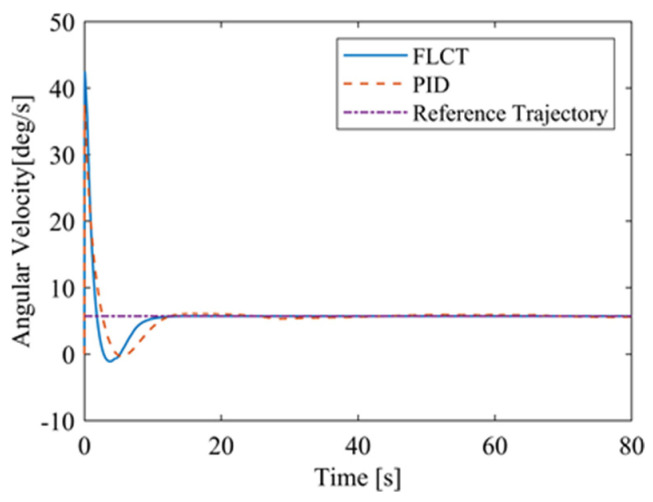
The angular velocity of the robot.

Figure 20 and Figure 21 show the velocity and the angular velocity of the robot with time. For the FLCT controller, the velocities stabilize after about 12.5 s, while the velocities fluctuate for the PID controller. This is because the wall-climbing robot is not in steady-state motion when it moves on a circular trajectory on the wall. The PID controller will only respond when the speed deviation occurs.

**Figure 22:**
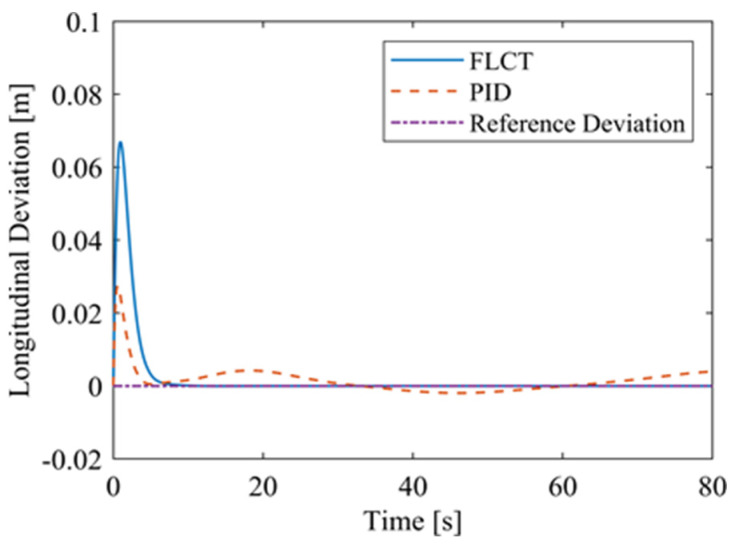
Longitudinal distance deviation.

**Figure 23:**
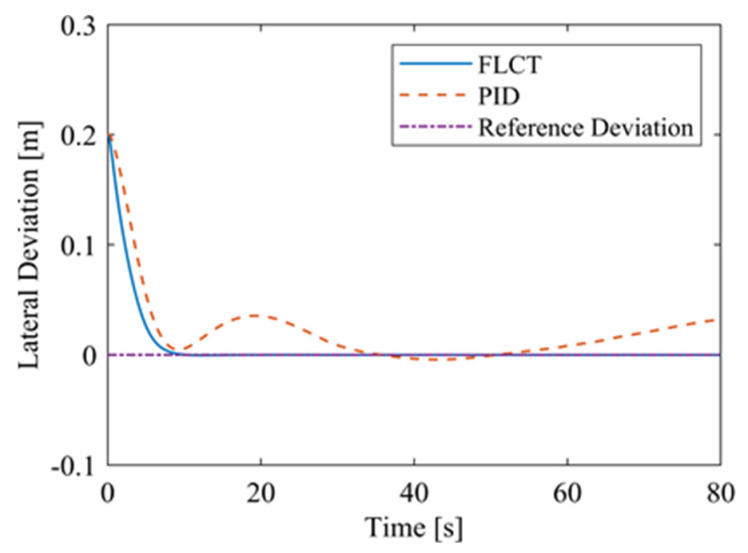
Lateral distance deviation.

**Figure 24:**
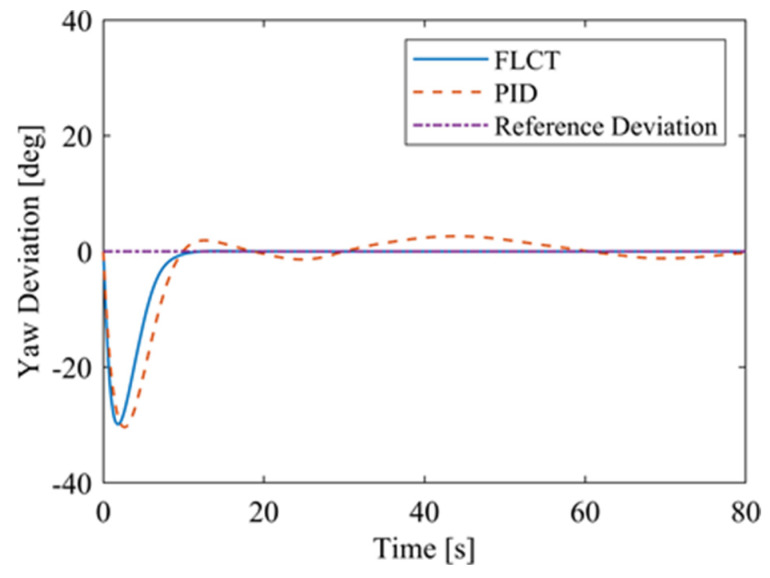
Yaw angle deviation.

**Figure 25:**
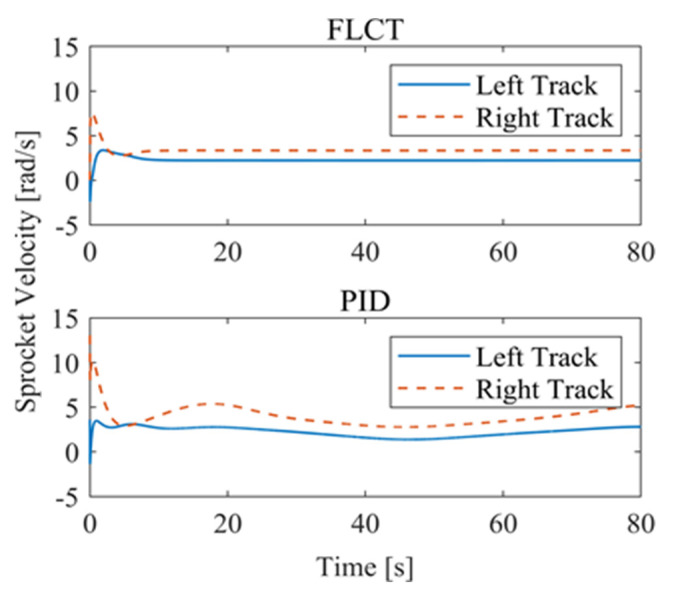
Sprocket velocities.

**Figure 26:**
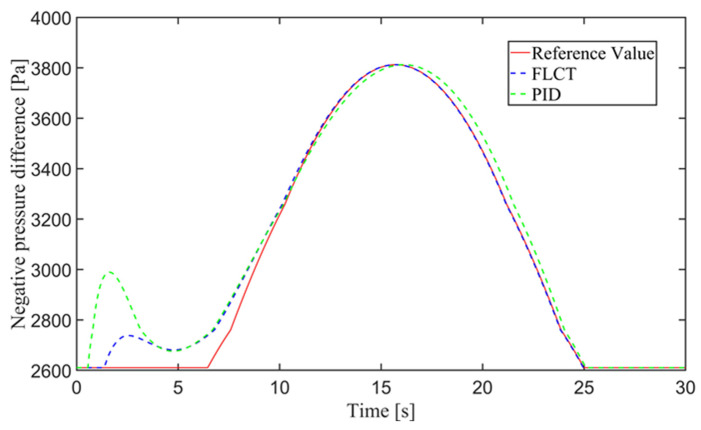
Negative pressure of adsorption unit.

For the FLCT controller, it can be seen from Figure 22 that the longitudinal distance deviation at the initial stage is 0, and the longitudinal distance deviation reaches the maximum at about 1.1 s, until it decreases to 0 at 12.5 s. It can be seen from Figure 23 and Figure 24 that the controller is mainly to reduce the lateral distance deviation at the initial stage. The robot quickly turns to the reference trajectory. Starting from 2 s, the robot approaches the reference trajectory and the effect of the yaw angle deviation increases, so that the robot gradually returns to the right, and finally, it is consistent with the reference trajectory. Since the PID controller does not consider the dynamics of the robot, it cannot be adjusted to give an appropriate control amount according to the motion state of the robot. When the pose of the robot changes, there will be a deviation. The angular velocity of the sprockets is shown in Figure 25. It can be seen that under the action of the FLCT controller, the velocity starts to converge at about 12.5 s, but it is not stable. This is because the circular motion of the robot is not stable; with the change in the pose of the robot, the controller output needs to be adjusted in a range to make the robot drive along a circular trajectory. The change in negative pressure in the adsorption system is shown in Figure 26; with the change in the robot’s position, the negative pressure system also adjusts the negative pressure value accordingly, and compared with the PID controller, the FLCT controller, when controlling the negative pressure value of the robot, has a smaller overshooting amount, a shorter time to reach the steady state and a smaller deviation value.

Comprehensive simulation results show that the FLCT control method proposed in this paper requires less rectification time to reach a stable control state compared with the traditional PID control method, both in trajectory-tracking control and negative pressure value control. After reaching the stable control state, the error values of the results of the FLCT control method are lower than those of the traditional PID method, indicating that the tracking effect of the proposed method and the negative pressure value control effect are more stable and accurate. However, in terms of the overshooting amount, FLCT does not show an advantage over PID, and there is a situation where the overshooting amount is larger than that of the PID method. One possible reason for this phenomenon is that the amount of overshooting is related to a variety of factors, such as the robot’s initial attitude and the given initial velocity. Another important reason is that the traditional PID method ignores the dynamics of the wall-climbing robot and the effect of gravity on the robot’s operation process. The tracked wall-climbing robot is a mechanical system with noncomplete constraints, and the noncomplete system is characterized by nonintegrable constraint relations, which cannot express the robot’s position in space based on the displacement of the drive components. The PID controller is a simple controller based on a kinematic model, and although the kinematic controller can simplify the tracking problem of the noncomplete constrained system, this is based on the assumption that no slippage occurs that does not correspond to the actual situation. Therefore, although the FLCT method does not show advantages in the initial phase of control, the results obtained have a smaller error due to the fact that the method is better able to deal with the nonlinear effects when confronted with a nonlinear dynamical model, as well as to take into account influences such as gravity. For wall-climbing robots, the proposed FLCT method is more advantageous than the PID method in practical operations, as it is more important to reach a stable operating state and control accuracy quickly in practical operations.

## 5. Practical Experiments

The purpose of the experiments is to verify the effect of the control system in practical applications and the robustness of the controller. The wall-climbing robot mainly includes an adsorption system, a walking mechanism and an environment sensing system. The adsorption system consists of a DC brushless motor, a radial centrifugal fan, a negative pressure chamber and a sealing device, which is driven by the motor to rotate the centrifugal fan, discharging the gas between the blades of the centrifugal fan to the outside side of the fan, while the sealing device restricts the leakage of the outside air from the outside to the negative pressure chamber. Under the action of the adsorption force, the robot is tightly attached to the wall, and the walking mechanism drives the robot to move on the wall. A UWB device is installed to measure position and velocity, and an IMU device is installed to measure yaw angular position and velocity. The UWB positioning system consists of a LinkTrack module launched by Nooploop, which is a PNTC (Positioning, Navigation, Timing and Communication) local positioning system based on UWB (Ultra-Wideband) communication technology. Under typical application scenarios, the 2D positioning accuracy can reach within 10 cm. The bandwidth is up to 3 Mbps, it supports distributed ranging and digital transmission and it can simultaneously support 40 tags and 120 base station positionings. The model of IMU is SC-INS-100D4, the measurement accuracy of pitch/roll angle is 0.8°, the measurement accuracy of swing angle is 2° and the resolution is 0.01°. The negative manometer adheres to the inside of the negative pressure chamber cavity, the pressure range of the negative pressure gauge is 300~1100 hPa and the accuracy can reach 0.2 Pa.

**Figure 27:**
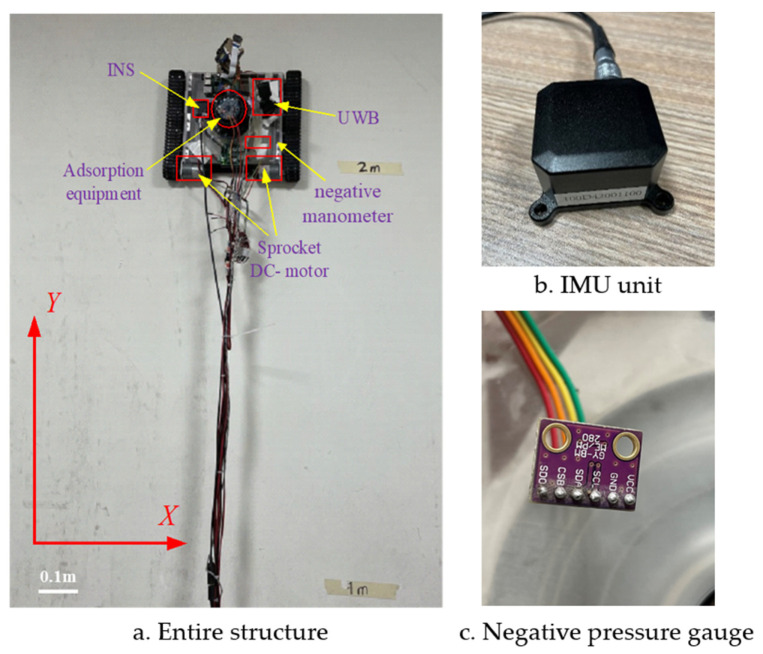
Experimental platform.

The wall-climbing robot used in the experiment is shown in Figure 27, and its parameters are shown in Table 2. The experiment was performed on a hard wall, i.e., the wall did not deform under the action of the robot.

### 5.1. Reference Straight Trajectory

**Figure 28:**
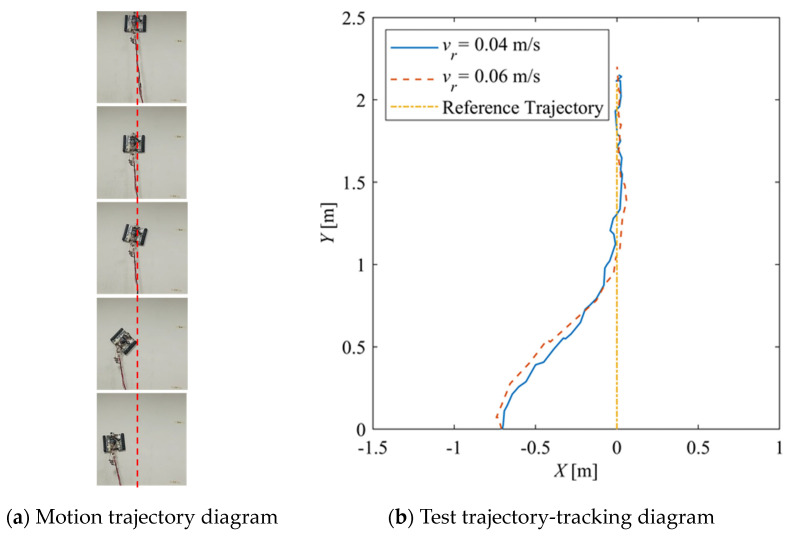
Straight-line tracking experiment trajectory.

Figure 28 shows the trajectories for which the reference velocities are *v_r_* = 0.04 m/s and *v_r_* = 0.06 m/s, respectively. The initial yaw angle deviation is 0°, and the initial position deviation is 0.7 m. It can be seen that for different reference velocities, the robot can quickly converge to the reference trajectory, and the final actual trajectory is consistent with the reference trajectory.

**Figure 29:**
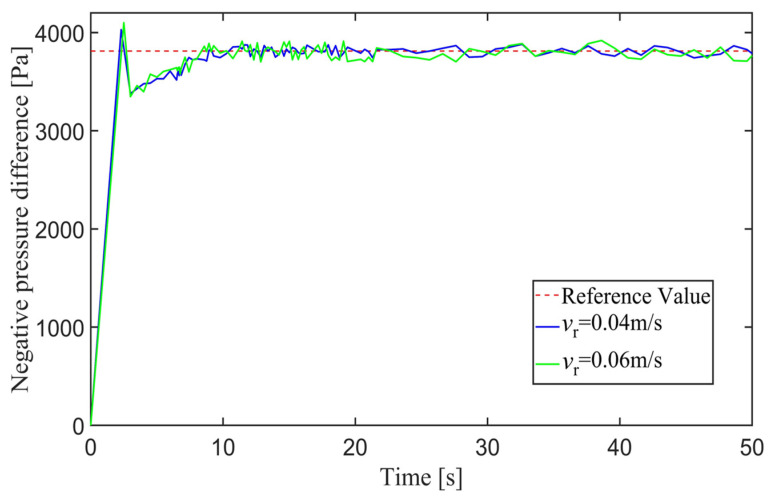
Negative pressure of adsorption unit.

Figure 29 shows the negative pressure value obtained by the controller when the robot is tracking a straight-line trajectory. At different tracking speeds, the robot is able to quickly converge on the negative pressure value, and the deviation of the negative pressure value gradually decreases close to 0 from the beginning of the robot’s movement and finally completes the stabilized control of the negative pressure value.

**Figure 30:**
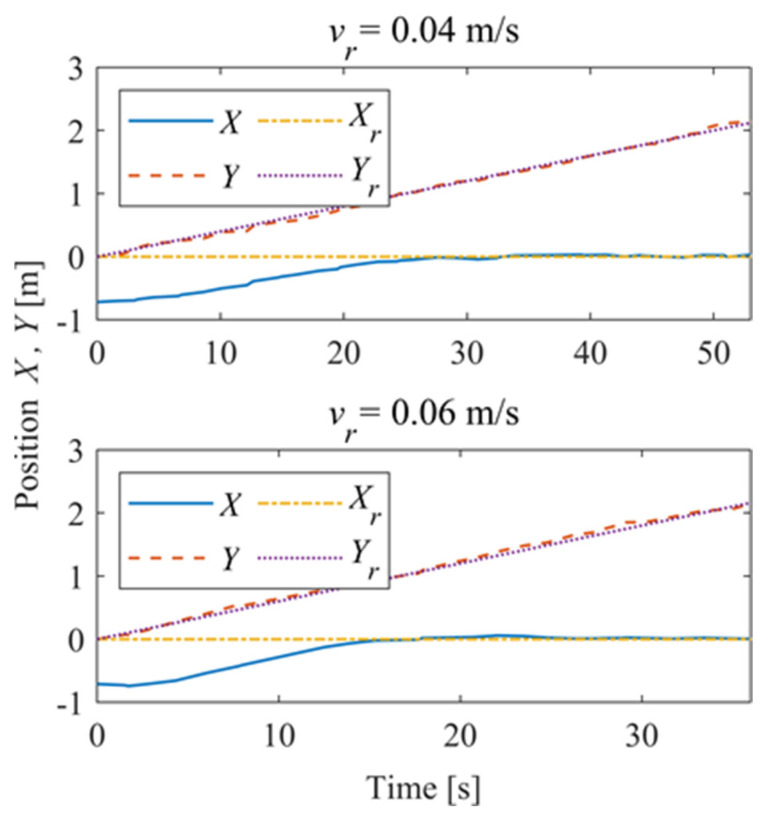
Position of the robot.

**Figure 31:**
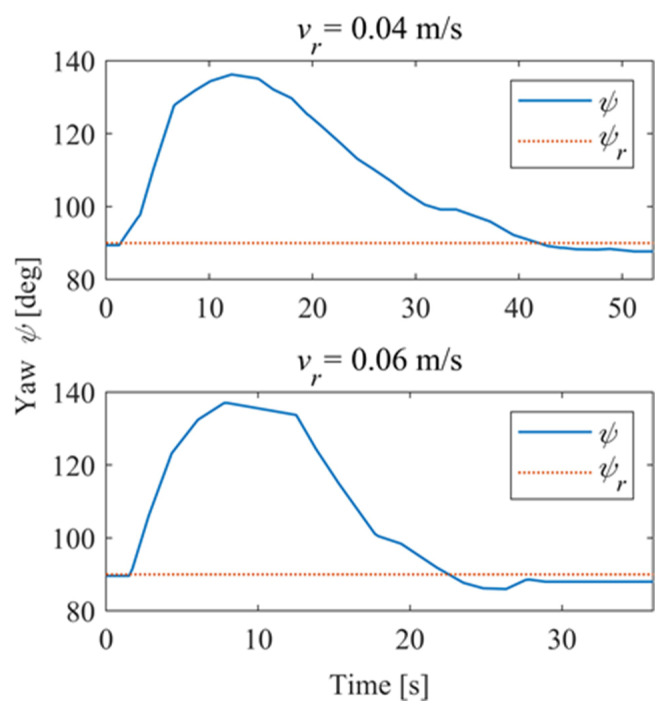
Pose of the robot.

Figure 30 shows the time–history of the position of the robot. For *v_r_* = 0.04 m/s, the deviation eliminates about 42 s and is stable; for *v_r_* = 0.06 m/s, the system is stable in about 28 s, and there is a slight overshoot. Figure 31 shows the time–history of the pose of the robot. A slight overshoot occurs when the reference velocity is high, and the tracking accuracy of the angle deviation can be stabilized within 3° after the system is stabilized.

### 5.2. Reference Curve Trajectory

**Figure 32:**
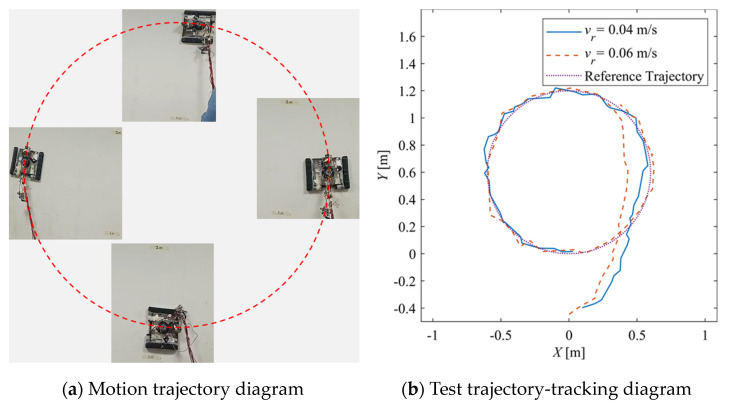
Curve tracking experiment trajectory.

**Figure 33:**
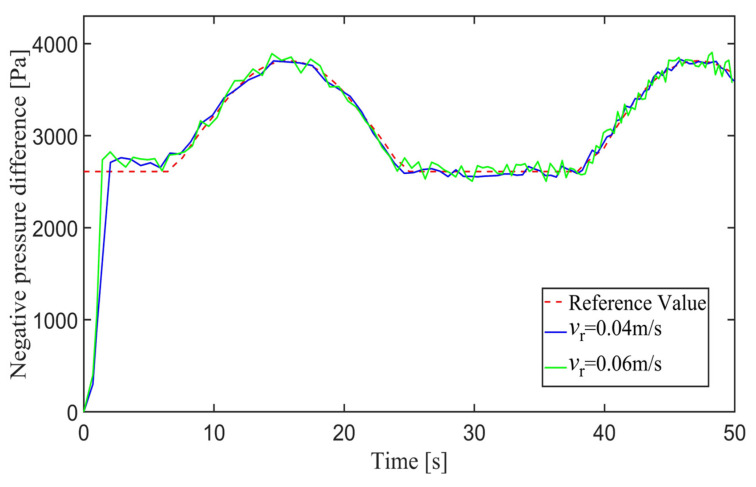
Negative pressure difference in adsorption units.

**Figure 34:**
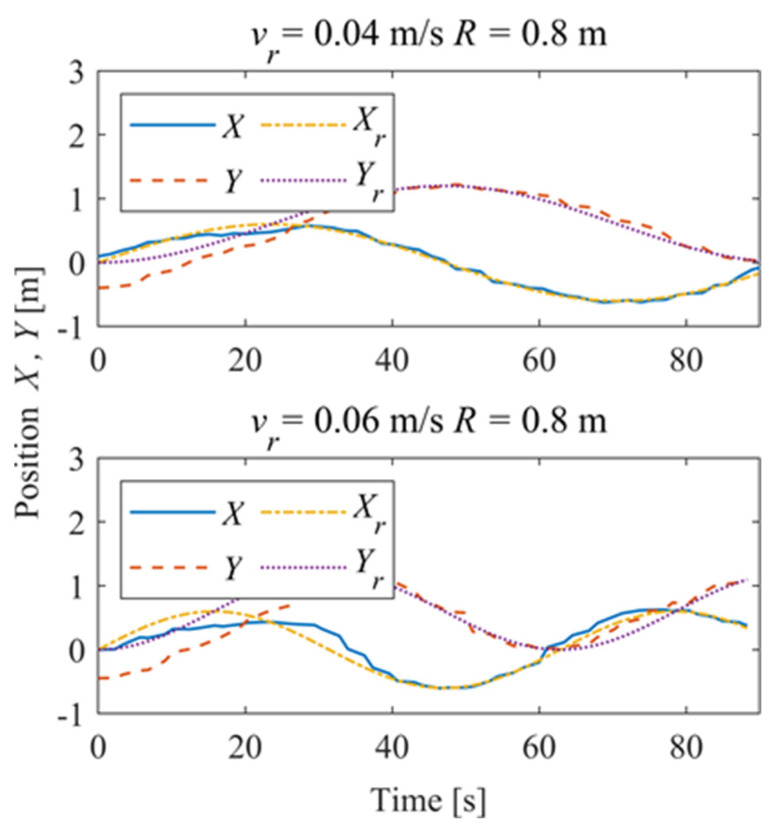
Position of the robot.

**Figure 35:**
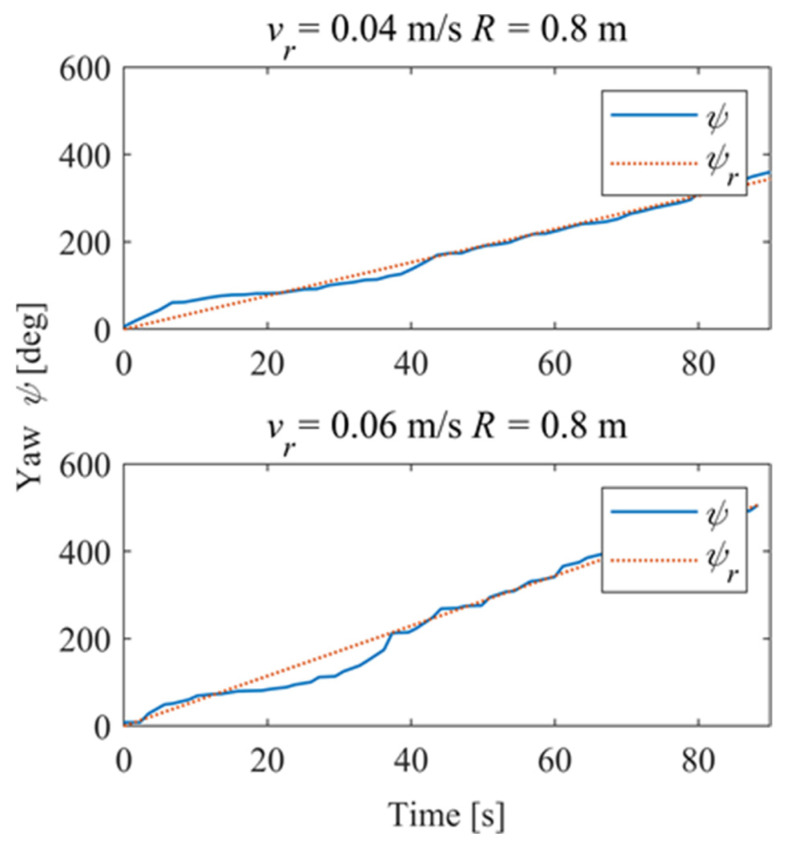
Pose of the robot.

In order to further explore the performance of the trajectory-tracking control system, several sets of experiments were carried out on curve driving conditions. Figure 32 shows the trajectories for which the reference velocities are *v_r_* = 0.04 m/s and *v_r_* = 0.06 m/s. It can be seen that under the curve conditions, the controller has better adaptability to different reference velocities. Figure 33 shows the negative pressure value obtained by the controller according to the current position of the robot. At different tracking speeds, the robot is able to adjust the negative pressure value quickly, and the deviation of the negative pressure value gradually decreases from the beginning of the robot’s movement, finally completing the stable control of the negative pressure value. When *v_r_* = 0.04 m/s, the robot’s negative pressure deviation is close to 0 in about 12 s, and when *v_r_* = 0.06 m/s, the robot’s negative pressure deviation is close to 0 in about 18 s. The faster the robot travels when tracking a circular trajectory, the faster the safety negative pressure value changes. Thus, compared with the robot moving at *v_r_* = 0.06 m/s, the negative pressure controller obtains a straight smoother negative pressure control and less deviation when the robot moves at *v_r_* = 0.04 m/s. Figure 34 is the time–history of the position of the robot. The system is stable at about 44 s for *v_r_* = 0.04 m/s, while the system is stable at about 39 s for *v_r_* = 0.06 m/s. Figure 35 shows the change in the yaw angle with time. When the reference speed is large, the overshoot of the yaw angle tracking is also relatively large, but because the driving speed is faster, it can converge to the reference trajectory earlier. The overshoot is relatively large when the reference velocity is large, but because of the faster velocity, it converges quickly to the reference trajectory.

## 6. Conclusions

In this paper, the authors proposed a trajectory-tracking and adsorption pressure control system for tracked wall-climbing robots based on a fuzzy logic computed-torque control method. First, based on the force analysis of the wall-climbing robot, a nonlinear dynamic model including nonholonomic constraints is established. Then, a trajectory-tracking controller based on the computed-torque control method and the negative pressure controller are designed. The parameters of the controller are tuned online based on the prescribed fuzzy rules to improve accuracy. In order to verify the effect of the proposed trajectory-tracking control system, several simulations and experiments are conducted. The simulation results show that the designed trajectory-tracking control system can effectively track the reference trajectory, and the designed negative pressure controller can quickly and accurately control the negative pressure value of the adsorption system. Compared with the classical PID controller, the proposed fuzzy logic computed-torque controller has better accuracy. The experimental results show that the control system is effective and robust in a practical application.

## Figures and Tables

**Table 1:** Fuzzy control rule table.

	$e_{y}$	NB	NM	Z	PM	PB
$e_{\psi }$						
NB		*K_y_*_1_/*K_ψ_*_3_/*K_p_*_3_	*K_y_*_2_/*K_ψ_*_2_/*K_p_*_4_	*K_y_*_3_/*K_ψ_*_1_/*K_p_*_5_	*K_y_*_2_/*K_ψ_*_2_/*K_p_*_4_	*K_y_*_1_/*K_ψ_*_3_/*K_p_*_3_
NM		*K_y_*_2_/*K_ψ_*_4_/K*_p_*_2_	*K_y_*_3_/*K_ψ_*_3_/*K_p_*_3_	*K_y_*_4_/*K_ψ_*_2_/*K_p_*_4_	*K_y_*_3_/*K_ψ_*_3_/*K_p_*_3_	*K_y_*_2_/*K_ψ_*_4_/*K_p_*_2_
Z		*K_y_*_3_/*K_ψ_*_5_/K*_p_*_1_	*K_y_*_4_/*K_ψ_*_4_/*K_p_*_2_	*K_y_*_5_/*K_ψ_*_3_/*K_p_*_3_	*K_y_*_4_/*K_ψ_*_4_/*K_p_*_2_	*K_y_*_3_/*K_ψ_*_5_/*K_p_*_1_
PM		*K_y_*_2_/*K_ψ_*_4_/*K_p_*_2_	*K_y_*_3_/*K_ψ_*_3_/*K_p_*_3_	*K_y_*_4_/*K_ψ_*_2_/*K_p_*_4_	*K_y_*_3_/*K_ψ_*_3_/K*_p_*_3_	*K_y_*_2_/*K_ψ_*_4_/*K_p_*_2_
PB		*K_y_*_1_/*K_ψ_*_3_/*K_p_*_3_	*K_y_*_2_/*K_ψ_*_2_/*K_p_*_4_	*K_y_*_3_/*K_ψ_*_1_/*K_p_*_5_	*K_y_*_2_/*K_ψ_*_2_/*K_p_*_4_	*K_y_*_1_/*K_ψ_*_3_/*K_p_*_3_

**Table 2:** Simulation Parameters of The Proposed Wall-climbing Robots.

**Robot parameters**	*m*	Mass	2.73 (kg)
	*Iz*	Inertia	0.0383 (kg·m^2^)
	*l*	Track contact length	0.28 (m)
	*B*	Tread of robot	0.3 (m)
	*H*	Height of robot center of mass	0.116 (m)
	*b*	Track width	0.045 (m)
	*l*_1_	Negative pressure chamber length	0.193 (m)
	*l*_2_	Negative pressure chamber width	0.179 (m)
	*d*	Sealing edge width	0.03 (m)
	*R*	Radius of the sprockets	0.027 (m)
	$K_{s}$	Negative pressure safety coefficient	2
**Wall parameters**	μ	Friction coefficient of the wall	0.57
	$\mu_{\delta }$	Sealing edge friction coefficient	0.28
	$\mu_{f}$	Rolling resistance coefficient	0.0263
	$\mu^{\prime }$	Lateral friction coefficient of the wall	0.388
	$\mu_{\delta }^{\prime }$	Sealing edge lateral friction coefficient	0.224
	γ	Wall inclination angle	0°
	*K*	Shear deformation modulus	0.01 (m)
**Kinematic parameters**	*V*_rl_	Straight tracking desired velocity	0.055 (m/s)
	*V*_rc_	Curve tracking desired velocity	0.075 (m/s)
	*l_s_*	Linear reference track length	0.5 (m)
	*d_c_*	Curve reference trajectory diameter	0.75 (m)

## Data Availability

Data are contained within the article.
